# Supplementary material for: Chitooligosaccharides Modulate Glucose-Lipid Metabolism by Suppressing SMYD3 Pathways and Regulating Gut Microflora
Source: Mar Drugs. 2020 Jan 20;18(1):69. doi: 10.3390/md18010069 (PMC7024377; doi:10.3390/md18010069)
Supplement: Supplementary file 1 [file marinedrugs-18-00069-s001.zip › STR Profiling Report/STR Profiling Report.pdf]

Cell Line Authentication Service

STR Profiling Report

Sample From: Tianjin University of Science and Technology

Sample Type: Cell Line

Testing Method: STR Genotyping

Report Time: July 31 2019

Add: Room 502, No.2, Zhongxing Creative Park, Lane 1015, Longteng Road, Songjiang District, Shanghai

COMPANY STATEMENT

1. THIS REPORT IS ONLY RESPONSIBLE FOR THE SAMPLES ANALYZED.
2. THE TESTING RESULTS AND THE ORGANIZATION NAME WILL NOT BE USED FOR ADVERTISEMENT, COMMERCIAL EXHIBITIONS, COMMERCIAL PERFORMANCE AND OTHER COMMERCIAL ACTIVITIES.
3. OBJECTIONS SHOULD BE RAISED WITHIN FIFTEEN DAYS AFTER THE RECEIPT OF THIS REPORT.
4. THE PAPER REPORT WITH CONTENT ALTERING, ADDING OR WITHOUT THE STAMPED SEAL OF THE COMPANY ARE INVALID.

Testing Company: Shanghai Biowing Applied Biotechnology Co. Ltd

Address: Room 205, NO.885 Wangjiashe Road, Songjiang District, Shanghai

Tel: +86-021-33559491

Contact: Wenyao Zhang

E-mail:market@biowing.com.cn

Cell Line Authentication – STR Profiling Report

Sample code

Table 1. Sample Code

Customer's code	Company Code
HepG2-3	20190731-01

Sample Number:1

Sample Type: Cell line

Testing Type: STR

Testing Method:

DNA was extracted by a commercial kit from CORNING (AP-EMN-BL-GDNA-250G). The twenty STRs including Amelogenin locus were amplified by six multiplex PCR and separated on ABI 3730XL Genetic Analyzer. The signals were then analyzed by the software GeneMapper.

Data Interpretation:

Cell lines were authenticated using Short Tandem Repeat (STR) analysis asdescribed in 2012 in ANSI Standard (ASN-0002) by the ATCC StandardsDevelopment Organization (SDO) and in Capes-Davis et al., Match criteria forhuman cell line authentication: Where do we draw the line? Int J Cancer.2013;132(11):2510-9.

Add: Room 502, No.2, Zhongxing Creative Park, Lane 1015, Longteng Road, Songjiang District, Shanghai

Test Results

1. STR profile

Table 2. STR and Amelogenin Genotyping Results of Cell line20191014-07.

Loci	Sampleinformation			Cell Bank information		
	Sample name：HepG2-3			Cell line name：HEP-G2		
	**Allele1**	**Allele2**	**Allele3**	**Allele1**	**Allele2**	**Allele3**
D5S818	11	12		11	12	
D13S317	9	13		9	13	
D7S820	10	10		10	10	
D16S539	12	13		12	13	
VWA	17	17		17	17	
TH01	9	9		9	9	
AMEL	X	Y		X	Y	
TPOX	8	9		8	9	
CSF1PO	10	11		10	11	
D12S391	21	25				
FGA	22	25				
D2S1338	19	20				
D21S11	29	31				
D18S51	13	14				
D8S1179	15	16				
D3S1358	15	16				
D6S1043	13	13				
PENTAE	15	20				
D19S433	15.2	15.2				
PENTAD	9	13				
D1S1656	11	12				

Add: Room 502, No.2, Zhongxing Creative Park, Lane 1015, Longteng Road, Songjiang District, Shanghai

2. database annotation

Figure 1. STR matching analysis

EV	Cell No.	Cell name	Locus names								
			D5S818	D13S317	D7S820	D16S539	VWA	TH01	AM	TPOX	CSF1PO
		Query (Your Cell)	*11,12*	*9,13*	*10,10*	*12,13*	*17,17*	*9,9*	*x,y*	*8,9*	*10,11*
1.00(36/36)	180	HEP-G2	11,12	9,13	10,10	12,13	17,17	9,9	X,Y	8,9	10,11

Note: The STR online match analysis of the test cell against DSMZ database, showing cell number (Cell No.) and cell name.

3. Authentication

☐ The submitted sample profile is human, but not a match for any profile in the DSMZ STR database.

☐ The submitted profile is an exact match for the following human cell line(s) in the DSMZ STR database (8 core loci plus Amelogenin):.

☑ The submitted profile is similar to the following DSMZ human cell line:HEP-G2.

- **Note:** A cell line can be considered to be authenticated when 80% (exact match) of the alleles in its STR profile match profiles from tissue or other cell line samples from that donor or from database. Cell lines with between a 55% to 80% (similar) match require further profiling for investigation of relatedness.

Add: Room 502, No.2, Zhongxing Creative Park, Lane 1015, Longteng Road, Songjiang District, Shanghai

Appendix:

1. Genotyping Strategy and Site Distribution

Table S1. Experimental Strategy and Sites

	Strategy 1	Strategy 2	Strategy 3	Strategy 4
1	D3S1358	D8S1179	D19S433	AMEL
2	VWA	D21S11	TH01	D1S1656
3	D7S820	D16S539	D13S317	D5S818
4	CSF1PO	D2S1338	TPOX	D12S391
5	PENTAE	PENTAD	D18S51	FGA
6			D6S1043	

The allele match algorithm compares the 8 core loci plus amelogenin only, even though alleles from all lociwill be reported when available.

2. DSMZ tools was used to carry on the cell line comparison, which contains 2455 cell lines STR data from ATCC, DSMZ, JCRB ,ECACC， GNE and RIKEN databases. If the cell is not included in the above cell library, users need to compared with other databases.

Technician: Jianan Zhang

Checked by: Chenqian Zhang

Issued by: Yang Bai

Issue date:July 31 2019

Add: Room 502, No.2, Zhongxing Creative Park, Lane 1015, Longteng Road, Songjiang District, Shanghai
