# Supplementary figures and images for: Chitooligosaccharides Modulate Glucose-Lipid Metabolism by Suppressing SMYD3 Pathways and Regulating Gut Microflora
Source: Mar Drugs. 2020 Jan 20;18(1):69. doi: 10.3390/md18010069 (PMC7024377; doi:10.3390/md18010069)

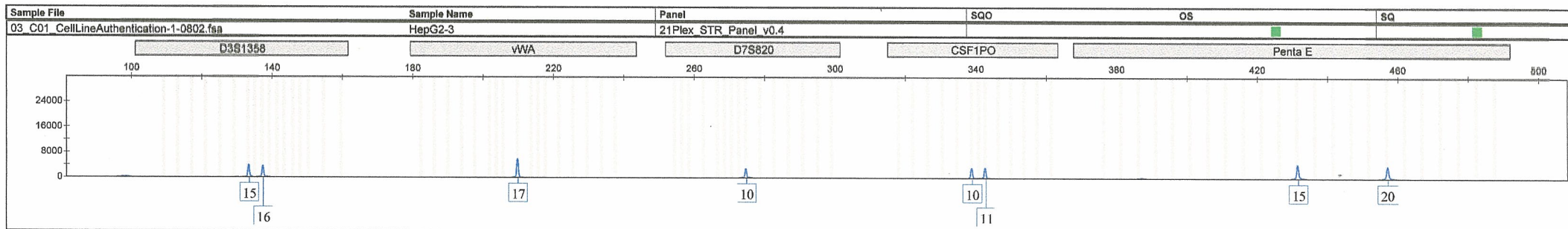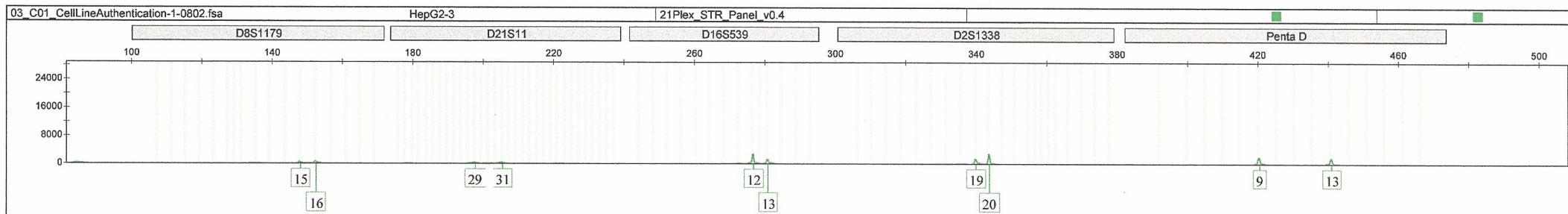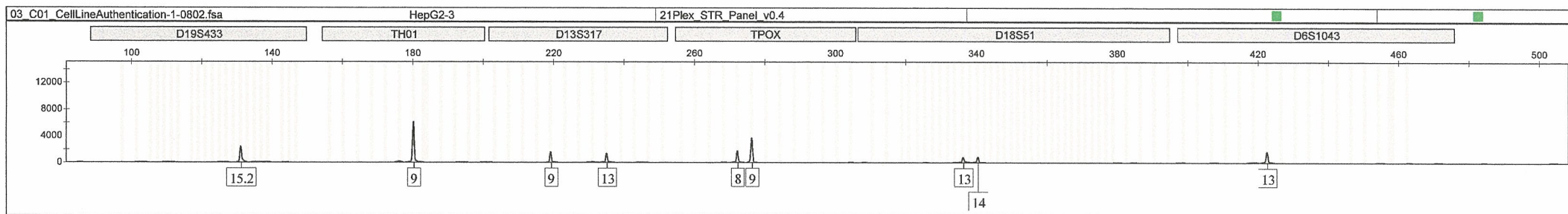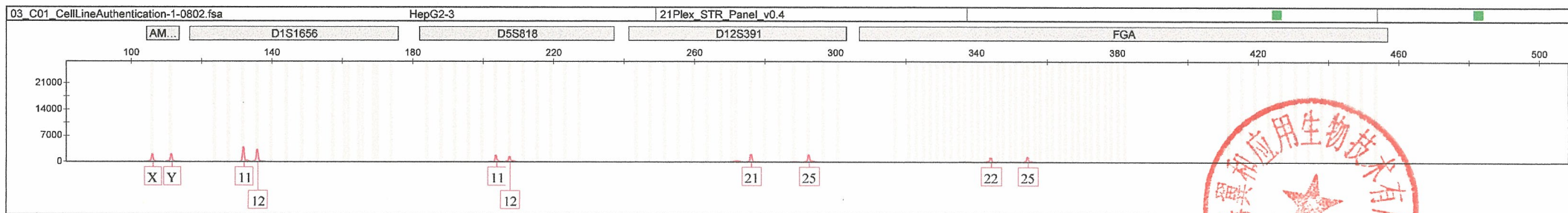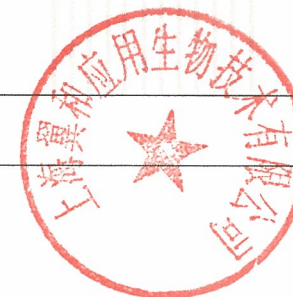

Supplement: Supplementary file 1 [file marinedrugs-18-00069-s001.zip › STR Profiling Report/GeneMapper.pdf]
